# Spatio-temporal decoupling of stomatal and mesophyll conductance induced by vein cutting in leaves of *Helianthus annuus*

**DOI:** 10.3389/fpls.2013.00365

**Published:** 2013-09-23

**Authors:** David T. Hanson, Laura E. Green, William T. Pockman

**Affiliations:** Department of Biology, University of New MexicoAlbuquerque, NM, USA

**Keywords:** stomatal conductance, leaf hydraulic conductance, mesophyll conductance, stomatal patchiness, chlorophyll fluorescence imaging, photosynthesis, transpiration, cavitation

## Abstract

Reduction of hydraulic conductance to the canopy has been shown to result in stomatal responses to limit transpiration. To test for similar responses to perturbations of the hydraulic network in leaves, we simultaneously measured leaf gas exchange with spatially explicit chlorophyll-*a* fluorescence and leaf temperature to examine the effects of cutting a primary leaf vein in *Helianthus annuus*. We repeated the leaf treatment at each of three different vapor pressure deficits and monitored the short-term dynamics of gas exchange following the treatment. Immediately after treatment, photosynthesis and stomatal conductance (*g*_s_) showed a transient “wrong way” response in which photosynthesis declined despite increased *g*_s_. Comparisons of fluorescence and temperature across the leaf showed that both photosynthesis and *g*_s_ were transiently patchy across the measured leaf area, but that the patchiness of the two processes did not correspond in space or time. This suggests that photosynthesis and *g*_s_ respond to vein cutting-induced cavitation via different mechanisms. Because the stomatal response varied by vapor pressure difference condition but photosynthesis did not, it is likely that *g*_s_, but not photosynthesis, responded to a hydraulic signal. In contrast, we hypothesize that photosynthesis declined due to a wound-induced electrical signal that has recently been shown to transiently decrease mesophyll conductance to CO_2_. The interaction of epidermal hydraulics and the electrical signal across the leaf likely created a patchy pattern of chlorophyll fluorescence and leaf temperature that cannot be explained through the action of a single signal.

## INTRODUCTION

In most terrestrial environments, plant growth and survival depends upon balancing water loss with CO_2_ fixation. Short-term regulation of water loss via transpiration (*E*) is controlled primarily by changes in stomatal aperture while changes in canopy leaf area (*A*_leaf_), leaf morphology, plant allometry, and other processes contribute to longer-term regulation. Photosynthesis (*A*) and *E* are generally correlated because stomatal aperture determines the conductance to both water vapor (*g*_s_) and CO_2_ (Farquhar and Sharkey, 1982). Therefore understanding the regulation of *g*_s_ is fundamental to understanding how plants balance water loss and CO_2_ uptake. The control of *g*_s_ is complex and is influenced by leaf water potential and transpiration rate in addition to other factors including CO_2_ concentration, light quality, and intensity (Mott and Parkhurst, 1991; Sperry and Pockman, 1993; Buckley, 2005; Messinger et al., 2006; Shimazaki et al., 2007). This study addresses how leaf hydraulic conductance influences the interaction between leaf water status, *g*_s_, *E*, and *A*.

Hydraulic conductance (*k*) is the flow rate of water per pressure difference across an entire plant or a portion of the flow path (Sack and Holbrook, 2006). As a result, *E* can be expressed in terms of hydraulic conductance from soil to leaf (*k*_whole plant_) and the difference between soil (ψ_soil_) and leaf (ψ_L_) water potential using an Ohm’s law analogy:

(1) $$E\times A_{leaf}=k_{whole\,\;plant}\times \left(\Psi_{soil}-\Psi_{L}\right)\,\;$$

*E* for a patch of leaf tissue is determined by *g*_s_ and the leaf-to-air vapor pressure difference (VPD). In the absence of stomatal regulation, increasing VPD will cause a proportional increase in *E* and decrease in ψ_L__,_ unless decreasing water potential causes a reduction of *k*_whole plant_ via xylem cavitation, decreased soil hydraulic conductance, or other mechanisms. Since hydraulic pathways through the leaf and root contribute most to whole plant hydraulic resistance (Yang and Tyree, 1994), *k*_leaf_ and ψ_L_ should be particularly important determinants of *g*_s_ and photosynthetic rate (*A*_net_; Sack et al., 2003; Sack and Holbrook, 2006).

Leaf vein size and architecture contribute to *k*_leaf_, but the spatial influence of any particular vein is hard to assess, partially because redundancy in vein networks and the relative importance of extra-xylary pathways for water movement are not well studied, though some significant efforts have been made (Nardini et al., 2001; Nardini and Salleo, 2003; Sack et al., 2008; Sommerville et al., 2010). As predicted by modeling studies (Tyree and Sperry, 1988), stomata respond to changes in *k* (Sperry and Pockman, 1993) and ψ_L_ (Saliendra et al., 1995), reflecting their integration of conditions up- and downstream in the soil–plant–atmosphere continuum. Correlations between *g*_s_ and *k* or ψ_L_ associated with xylem cavitation thresholds have been demonstrated (Sperry and Pockman, 1993; Saliendra et al., 1995; Mencuccini and Comstock, 1999; Salleo et al., 2000), but observations of seemingly contradictory stomatal responses have led some to argue that stomata also respond quickly to a hydraulic cue before leaf water status is negatively affected (e.g., Meinzer, 2002). The time frame over which these relationships are observed is important in understanding a potential signal, since Δψ oscillations that trigger stomata may be small, local, or transient, further confounding the relationship between *g*_s_ and ψ_L_ (Sperry et al., 1993; Saliendra et al., 1995). In addition, stomata may behave heterogeneously across leaves, but little is known about what initiates this phenomenon (Lange et al., 1971; Eckstein et al., 1998; Mott and Buckley, 1998; West et al., 2005).

Oscillating heterogeneity in *g*_s_ has been observed following changes in VPD, suggesting that guard cells are sensitive to small variations of water potential across leaves and that some level of interaction occurs among groups of leaf cells. This “patchy” behavior is thought to occur as neighboring cells interact with guard cells and transiently affect turgor pressure (Mott and Franks, 2001). Heterogeneous, small-scale stomatal responses might allow finer tuning of water balance even if the perturbation is large. Small, transient adjustments in stomatal aperture (and local cell ψ) would be undetectable in net measurements of ψ_L_, *E*, and *g*_s_ and potentially give the impression that ψ_L_ was controlled by some other mechanism (Saliendra et al., 1995; Lawson et al., 1998; Nardini and Salleo, 2003). Since leaf water balance is a function of both supply and demand (Lange et al., 1971), patchiness observed following changes in VPD (Mott and Franks, 2001; West et al., 2005) might be attributable to a sudden heterogeneous change in *k* caused by local leaf vein cavitation (Terashima, 1992). A more rapid step change in ψ of cells caused by sudden loss of conductance in a vein directly supplying them with water might possibly induce more dramatic patchy behavior. Some loss of xylem function may be tolerated to optimize gas exchange (Jones and Sutherland, 1991; Sperry et al., 1993; Mencuccini and Comstock, 1999), and may cause a drop in leaf cell water potentials resulting in initiation of stomatal closure. However, Nardini et al. (2001) found laurel leaf hydraulic architecture to be redundant and water to move through the leaf in parallel pathways, rather than in series. In this case, water could easily bypass a cavitated vein making the effect on stomata and gas exchange minimal or temporary.

We assessed the effect of altering hydraulic architecture by measuring spatial and temporal changes in *g*_s_, *E*, and *A* in *Helianthus annuus* after cutting a vein to simulate cavitation. Our initial hypothesis was that the reduction of *k* to tissues predominantly supplied by the cut vein would decrease local ψ_L_ leading to stomatal closure. However, we observed an apparent transient patchy stomatal response based on patchy chlorophyll fluorescence that demonstrated a transient disconnect between *A* and *g*_s_ not predicted by existing models. In addition, local *A* and *g*_s_ returned to near initial levels despite the severing of the primary leaf vein. This transient response differed from prior studies of patchy stomatal behavior because it was easily reproduced and because the patchy stomatal conditions were associated with a transient “wrong way” response to wounding where *A* decreases despite increases in *g*_s_. These observations suggested a different mechanism for inducing patchy stomatal behavior than West et al. (2005) described for *Xanthium strumarium* despite the similar heterobaric anatomy and compartmentation of mesophyll found in both *H. annuus* and *X. strumarium* (McClendon, 1992; Pieruschka et al., 2005). In order to understand this unexpected complexity in the interaction between hydraulic architecture, *g*_s_, and *A* we added measurements of spatial changes in leaf temperature to our measurements of gas exchange and chlorophyll fluorescence imaging and measured the response under three VPDs. Our results highlight that the mechanism for varying leaf water potential can have large and unexpected impacts on the observed relationship between hydraulic conductance, stomatal behavior, and gas exchange that could lead to erroneous mechanistic models.

## MATERIALS AND METHODS

### PLANT MATERIAL

*Helianthus annuus* seeds were germinated and grown in a Conviron growth chamber (Winnipeg, Manitoba, CA, USA) for 25–30 days where they received 12 h of 500 μmol m^-^^2^ s^-^^1^ light per day. Relative humidity was ^~^50% and temperature was controlled at 23°C during the dark period and 27°C during the light period. Plants were fertilized three times per week with Jacks water soluble 20–20–20 (N–P–K) and were well watered.

### TREATMENT

With the entire plant inside the growth chamber, a 2 cm × 2 cm area of one fully expanded leaf per plant, including the primary vein 1 cm from petiole, was enclosed in a gas-exchange cuvette (LiCor 6400, Lincoln, NE, USA) where air temperature was maintained at 25°C and reference CO_2_ was constant at 400 ppm. The flow rate through the chamber was 500 μmol s^-^^1^ so the time constant of the chamber was approximately 5 s. A fluorescence camera was attached to a custom 2 cm × 2 cm cuvette (Leaf Chamber FluorCam, Photon Systems Instruments, Ltd., Czech Republic) to provide spatially explicit measurements of chlorophyll-*a* fluorescence. For comparison of spatial stomatal behavior with fluorescence imaging, a custom-built thermocouple array made up of 13 evenly spaced copper-constantan thermocouples (36 gage, 0.127 mm diameter, Omega), in contact with the abaxial side of the leaf and measured by a datalogger (Campbell Scientific model BR7, Logan, UT, USA), was used to measure spatial changes in leaf temperature.

Each leaf was dark adapted for 20 min, after which the quantum efficiency of open photosystem II (PSII) centers (*F*_v_/*F*_m_) was measured using a saturating flash and measuring light (PAR = 0.03 μmol quanta m^-^^2^ s^-^^1^). Saturating flash intensity was varied in trial experiments (during dark and light) to ensure the intensity used was sufficient to saturate PSII (data not shown). The blue (peak = 450 nm) and red (peak = 628 nm) actinic lights of the fluorescence camera were then turned on to a level that matched the light intensity in the growth chamber (approx. 500 μmol quanta m^-^^2^ s^-^^1^) outside of the cuvette (50% each red and blue). The leaf was allowed to reach steady state photosynthesis (±0.5 μmol m^-^^2^ s^-^^1^) and transpiration (±0.5 mmol m^-^^2^ s^-^^1^) for 10 min before a saturating pulse was applied to determine the quantum yield of PSII photochemistry (Φ_PSII_). The leaf was again allowed to reach steady state following the saturating pulse, at which time the fluorescence camera measuring light was turned on so that fluorescence in the light (*F*′) was measured every 5 s for 15 min which was sufficient to capture the dynamic responses. Over short time periods on a single leaf area and single light intensity, changes in *F*′ are directly proportional to 1 - *F*′/F’_m_ (see West et al., 2005). Simultaneously, measurements of net CO_2_ and H_2_O exchange were stored every 5 s and thermocouple temperatures were recorded every 1 s. A cut was made through the primary leaf vein just outside the cuvette, 1 cm from the petiole and the junction between the main vein and the two secondary veins, without damaging surrounding leaf tissue. Fluorescence, and thermocouple temperature data were logged from steady state to 900 s following the cut while gas exchange data continued to be logged until 30 min following the cut. After 30 min, a second Φ_PSII_ measurement was taken. This protocol was repeated six times for each of three reference VPD treatments: 2, 1.25, and 0.5 kPa corresponding to approximately 15, 40, and 70% relative humidity. Reference humidity was controlled manually using the LI-6400 desiccant.

### DATA ANALYSIS

Pixels within a 2 mm radius of the estimated position of each thermocouple were averaged and used to spatially compare the *F*′ response with temperature response. These 13 circular areas are referred to as “sub-areas.” The average *F*′ of these sub-areas is used as average leaf *F*′ when compared to average leaf temperature (the average of the 13 discrete areas). When average *F*′ response was compared to net gas exchange, whole leaf *F*′ averages (all pixels included) were used. Net *g*_s_ was calculated using the average temperature for all thermocouples. Spatial heterogeneity of fluorescence and temperature within each leaf was estimated by calculating standard deviation of fluorescence/temperature in each of the 13 leaf sub-areas at four time intervals. The time intervals were defined as: (I) before the cut, (II) at the peak of the response (time of highest *F*′ or lowest temperature), (III) at 300 s, and (IV) at 900 s. Heterogeneity of recovery time was estimated by calculating standard deviation of peak response times (time at which parameter changed direction) in each of the 13 leaf sub-areas. Parameter and VPD treatment means were compared using two-way *t*-tests with unequal variance.

## RESULTS

### WHOLE LEAF RESPONSE

**FIGURE 1:**
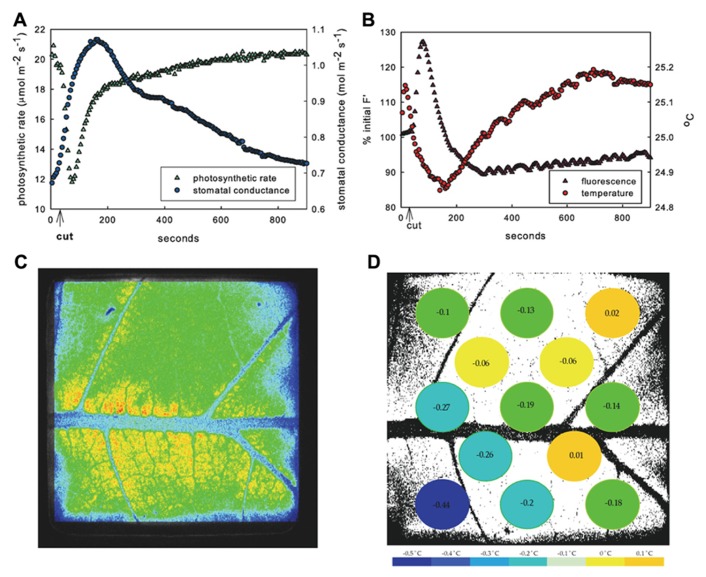
**Response of example leaf in 1.25 kPa VPD treatment.** Mean response to leaf vein cut in entire measured leaf area of photosynthesis (green triangles) and stomatal conductance (blue circles) **(A)** and fluorescence (purple triangles) and temperature (red circles) **(B)** showing typical inverse relationship of photosynthetic rate and stomatal conductance. Image of fluorescence **(C)** and temperature in 13 leaf sub-areas **(D)** temperature change (°C) at peak time of average fluorescence response following cut.

Across all VPD treatments, cutting the main leaf vein initiated transient opposite responses in net carbon assimilation (*A*) and transpiration rate. Typical leaves responded to treatment with an immediate and rapid decrease in *A* and a simultaneous *increase* in *E* and calculated stomatal conductance (*g*_s_; **Figure 1A**). The average decrease in *A* was 8.5 ± 4.1 μmol CO_2_ m^-^^2^ s^-^^1^ to ~40% of steady state, while the average increase in *E* was 1.3 ± 1 mmol H_2_O m^-^^2^ s^-^^1^ which was near the ~20% maximum increase from steady state, and *g*_s_ increased 0.22 ± 0.23 mol H_2_O m^-^^2^ s^-^^1^ across VPD treatments (**Table 1**). The increase in *E* is consistent with an increase in *g*_s_, suggesting that the observed decline in photosynthesis was not the result of a CO_2_ limitation that might occur with decreased *g*_s_. The response of *A* and *E* were not synchronous, with minimum *A* preceding maximum *E* in all leaves across all VPD treatments (e.g., **Figure 1A**). Photosynthesis reached its minimum an average of 64 ± 11 s after the cut and *E* reached its highest rate significantly later (*p* = 0.0006), at an average of 143 ± 80 s after the cut (estimated *g*_s_ reached its highest rate 156 ± 78 s after the cut) and varied with VPD treatment.

**Table 1:** Effect of VPD on the responses to vein cutting.

	Steady state			Maximum change from steady state		
VPD	*A* (μmol m^-2^ s^-1^)	*E* (mmol m^-2^ s^-1^)	*g*_s_ (mol m^-2^ s^-1^)	*A* (μmol m^-2^ s^-1^)	*E* (mmol m^-2^ s^-1^)	*g*_s_ (mol m^-2^ s^-1^)	*T* (°C)
0.5	21.6 ± 3.1^a^	7.3 ± 0.9^a^	1.2 ± 0.3^a^	-7.8 ± 5.2^a^	0.3 ± 0.7^a^	0.3 ± 0.4^a^	-0.14 ± 0.09^a^
1.25	21.6 ± 2.7^a^	8.8 ± 0.8^b^	0.6 ± 0.1^b^	-8.9 ± 1.3^a^	1.9 ± 0.9^b^	0.3 ± 0.2^a,b^	-0.26 ± 0.12^a^
2.0	18.4 ± 3.2^a^	10.8 ± 3.2^c^	0.4 ± 0.1^b^	-8.9 ± 5.3^a^	1.7 ± 0.8^b^	0.1 ± 0.2^a,c^	-0.21 ± 0.19^a^

*Errors are standard deviations and letters in superscripts are different if differences between VPD treatments are significant at the *p* = 0.05 level.*

### SPATIAL VARIATION ACROSS LEAF

**FIGURE 2:**
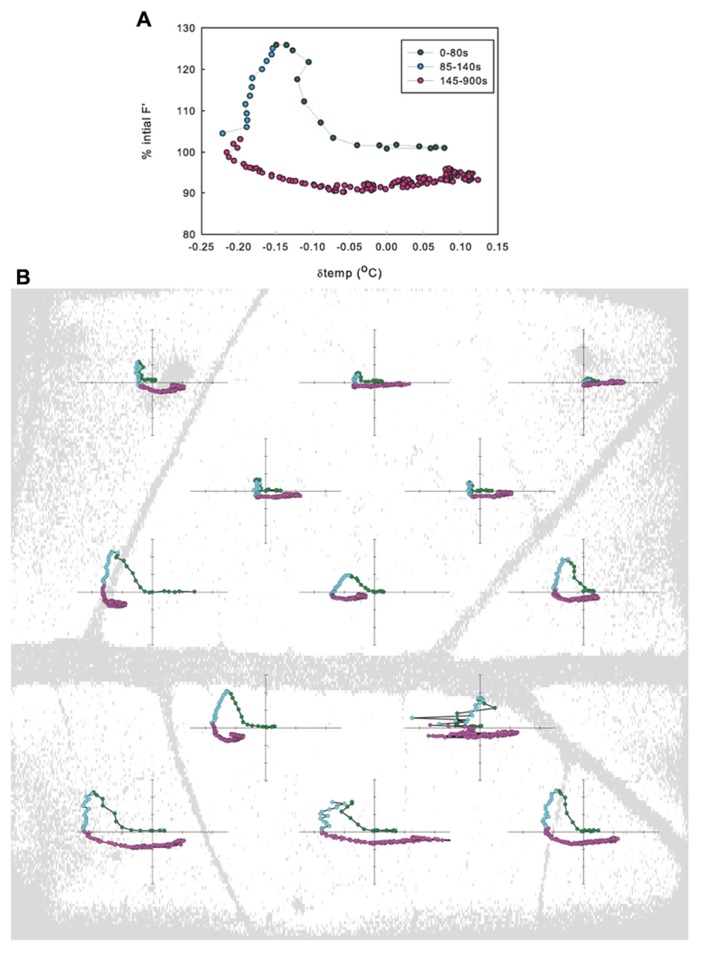
**Relationship between the change in fluorescence (Δ_F__′_) and the change in temperature (Δ_temp_) when averaged across a leaf (A) and when averaged in leaf sub-areas (B) for example leaf in 1.25 kPa VPD treatment**.

The decrease in *A* and increase in *E* appeared immediately upon cutting the vein. Both photosynthetic and stomatal responses displayed spatial heterogeneity as measured by fluorescence imaging and variation of temperature across the thermocouple array (**Figures 1C, D** and **2**). However following initiation, *A* and *E* changed at different rates and the spatial pattern of the response differed between parameters. The peak fluorescence response preceded the peak temperature response in all areas of the leaf by an average of 82 s, although the time by which the extremes were separated varied across the leaf (mean standard deviation of the separation across leaf sub-areas was 55 s). Photosynthetic heterogeneity of leaf sub-areas increased significantly following the cut (*p* < 0.01) and returned to pre-cut variability by 300 s, but heterogeneity of *T*_leaf_ did not significantly increase in response to the cut (not shown), although temperature did respond differentially across the leaf (**Figures 1D** and **2**). While often the same general area of a leaf saw the greatest overall changes in both *F*′ and temperature, at the time of peak fluorescence (65 ± 10 s following cut), many sub-areas where a decrease in electron transport (approximated by an *F*′ increase) was observed did not show evidence of stomatal closure (i.e., temperature increase; **Figures 1C, D** and **2**). In many areas where *F*′ increased a there was also a decrease in temperature, reinforcing the transient inverse relationship between measured net rates of photosynthesis and *g*_s_. However, some sections of the leaf saw changes in *F*′ with no corresponding change in temperature or vice versa.

No significant differences were found between Φ_PSII_ or *F*_v_/*F*_m_ values across treatments either before leaf vein cuts or after photosynthesis had recovered post vein cutting, indicating that biochemical adjustments (non-photochemical quenching) were probably not a factor. *F*_v_/*F*_m_ averaged 0.80 ± 0.02; Φ_PSII_ before cut averaged 0.51 ± 0.02; Φ_PSII_ 30 min after cut averaged 0.50 ± 0.02. Most leaves responded with what could be described as a three-phase response: (1) 0 to ~80 s photosynthetic minimum, in which *A* and *E* are inversely related, in most cases, (2) ~85 to ~140 s start of *A* recovery and peak of transpiration response (*A* and *E* are directly related), and (3) ~145–900 s start of *E* recovery (*A* and *g*_s_ are again inversely related, but the nature of the relationship varies widely between leaves; **Figure 2A**). Although individual leaf sub-areas also exhibited the three-phase response characteristic to the net gas exchange response, no consistent relationship between temperature and fluorescence was found across the leaf, suggesting that net relationships observed in entire measured leaf areas are not representative of smaller scale responses (**Figure 2B**).

### VPD TREATMENT COMPARISONS

#### Steady state

At steady state (pre-cut), *E* increased significantly with increasing VPD, therefore leaves in higher VPD treatments most likely experienced lower water potentials. *g*_s_ decreased significantly with increasing VPD which may have contributed to the small decrease in *A* at high VPD (**Table 1**).

#### Maximum response

**FIGURE 3:**
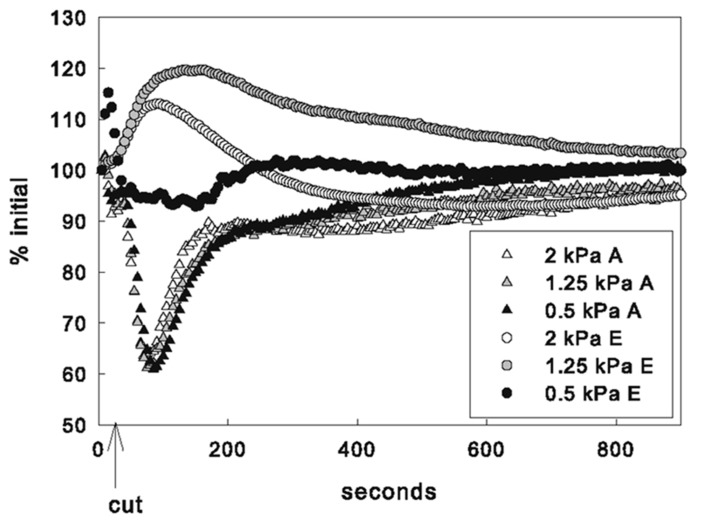
**Mean response of gas exchange by VPD treatment as % of initial (steady state) values**.

No significant differences in the average decrease in *A* or increase in average *F*′ (not shown) were detected among VPD treatments (**Table 1**). Increases in mean *E* were significantly higher for leaves in both the 2 and 1.25 kPa VPD treatments than leaves in the 0.5 kPa VPD treatment (**Figure 3**, *p*-value = 0.04 and 0.008, respectively). The *E* response varied widely for leaves in the 0.5 kPa treatment where the average *E* increase was not statistically different from 0. The 1.25 kPa VPD treatment averaged the greatest transpiration rate increase among humidity treatments, so average *E* increase in response to the cut did not vary proportionally with VPD treatment. The difference between the *E* increase in the 1.25 and 2 kPa treatments was not significant. However the average estimated *g*_s_ increase for leaves in the 1.25 kPa treatment was found to be significantly greater (*p* = 0.05) than the average 2 kPa *g*_s_ increase. The average leaf temperature also followed this pattern, although no differences in temperature between VPD treatments were found to be statistically significant.

#### Initiation of recovery

Across VPD treatments, there were no differences in the average timing of the minimum photosynthetic rate, as measured by either gas exchange or fluorescence. In contrast, the time it took the transpiration rate to reach its peak following the cut increased with decreasing VPD. *E* peaks were reached at 76 ± 41, 113 ± 31, and 239 ± 41 s after the cut for the 2, 1.25, and 0.5 kPa VPD treatments, respectively. Average *E* peaks of leaves in the 0.5 kPa VPD treatment were significantly later than both the 2 and 1.25 kPa VPD treatments (*p* = 0.00004 and 0.0003, respectively). No significant differences between average time of lowest average leaf temperature were found between treatments, although they followed a similar pattern.

No significant differences in average fluorescence heterogeneity were found between VPD treatments at any time before or after the cut (not shown), but treatments did differ in temperature heterogeneity. Temperature heterogeneity of leaves in the 1.25 kPa VPD treatment was often significantly lower than that of leaves in other VPD treatments at the same times. Significant differences in temperature heterogeneity were found between VPD treatments 1.25 and 0.5 kPa VPD treatments before leaf vein cut (*p* = 0.012), between 1.25 and both 2 (*p* = 0.034) and 0.5 kPa (*p* = 0.036) VPD treatments at the peak of the response, and between 1.25 and 0.5 kPa VPD treatments at both 300 (*p*-value = 0.007) and 900 s (*p-*value = 0.026).

### Heterogeneity of recovery initiation

Much more variability in the times of peak responses was found in temperature than in fluorescence across each leaf, demonstrating that the initiation of photosynthetic recovery was more temporally coordinated than that of the stomatal response. The average standard deviation of the timing of maximum fluorescence in 13 sub-areas averaged across all leaves was 8 ± 3 s, while the standard deviation of the timing of minimum temperature for these same sub-areas averaged 32 ± 11 s, a significantly different spread (*p* = 0.00001). Standard deviations of times of temperature lows (interpreted as just before stomatal aperture began to decrease) across the leaf averaged 40 ± 26, 35 ± 15, and 24 ± 8 s for 2, 1.25, and 0.5 kPa VPD treatments, respectively. The fluorescence response recovery time varied very little with VPD treatment. Standard deviations of fluorescence response peak times were 5 ± 2, 7 ± 4, and 8 ± 2 s for 2, 1.25, and 0.5 kPa VPD treatments, respectively.

#### Recovery

By 900 s, all leaves had recovered and gas exchange rates were at or near pre-cut values regardless of VPD treatment. No significant differences in gas exchange recovery rates were detected between VPD treatments at 300 s. Although, by 900 s, the average *A* in leaves in the 0.5 kPa VPD treatment of 100.2 ± 2.9% pre-cut rate was significantly higher (*p* = 0.01 and 0.02 for 2 and 1.25 kPa VPD treatments, respectively) than that of other treatments (92.2 ± 5.04 and 95.8 ± 2.8% pre-cut *A* in 2 and 1.25 kPa VPD treatments, respectively).

## DISCUSSION

Cutting a primary leaf vein to simulate cavitation caused transient “wrong way” responses in transpiration and photosynthesis in the region of the leaf closest to the affected vein (**Figures 1** and **3**). Concurrent spatially explicit measurements of leaf temperature and chlorophyll-*a* fluorescence showed that the responses measured by gas exchange were the net result of underlying variation across the leaf, where the inverse response of photosynthesis and *g*_s_ was common in many but not all leaf sub-areas (**Figure 2**). This pattern of transient increases in transpiration and concomitant decreases in photosynthesis was consistent across measurements at three levels of VPD. These data suggest that the increase in transpiration was due to changes in water potential following the cut but that the photosynthetic response reflects a non-stomatal limitation triggered by the treatment. Fully demonstrating the basis of the non-stomatal limitation of assimilation will require more detailed analysis of magnitude and spatial extent of water potential changes and action/variation potentials (VPs) triggered by the treatment. However, a simple explanation is offered by Gallé et al. (2013) that found a similar inverse response of *A* and *E* to wounding (burning of an adjacent leaflet). They showed that the decrease in *A* was due to a concurrent drop in mesophyll conductance to CO_2_ (*g*_m_) triggered by a wound-induced electrical signal.

### TRANSIENT WRONG WAY A AND E RESPONSE: HETEROGENEITY

Chlorophyll-*a* fluorescence imaging has been used as a non-destructive way to detect and record dynamics of heterogeneous behavior across the leaf that cannot be measured by gas exchange methods. Previous studies have shown that uneven increases in fluorescence images can be caused by CO_2_ limitation resulting from stomatal closure since areas of increased *F*′ values correlated with increased temperature following changes in VPD (West et al., 2005). In the present study, however, non-uniform *g*_s_ in response to vein-cutting was not correlated with stomatal limitation of *A*. At the same time that *A* was decreasing, *g*_s_ increased significantly and at other times *A* increased while *g*_s_ decreased. Independent spatially explicit measurements of leaf temperature indicated that temperature decreased in many leaf sub-areas where fluorescence increased, although no consistent relationship (negative or positive) between the magnitude of the responses was found (**Figures 1C, D**). Some areas of the leaf changed temperature with little change in fluorescence, whereas in other areas fluorescence increased and decreased while temperature was dropping (**Figure 2**). Additionally, VPD had no effect on the response of *A* to the cut but did affect the magnitude and timing of the *E* response (**Figure 3**), indicating a hydraulic component in the response and recovery of stomata that was not evident in the photosynthetic response.

### TRANSIENT WRONG WAY A AND E RESPONSE: SUDDEN DECREASE IN WATER POTENTIAL

Stomatal conductance increased immediately following the treatment, indicating that average stomatal aperture increased. This change occurred faster than osmotic potentials could change actively, and because the manipulation of the hydraulic architecture supplying the measured leaf area occurred at the leaf, it is unlikely that signals from the subtending stem or roots played a role. Therefore the most likely explanation for the stomatal response is a passive effect of sudden ψ changes produced by the cut. A similar transient increase in *E* and *g*_s_ has also been observed in experiments in which a whole leaf was excised at the petiole. Described as the transient “wrong way” stomatal response (Buckley, 2005), it is thought to be an effect of sudden loss of turgor pressure in subsidiary epidermal cells, releasing pressure on guard cells and increasing aperture (Darwin and Pertz, 1911; Iwanoff, 1928; Willis et al., 1963; Raschke, 1970). Raschke (1970) described a stomatal response on a time scale similar to that observed in the present study in which a decrease in xylem ψ of *Zea mays* was transmitted to stomata in 0.1 s, causing *g*_s_ to increase. Willis et al. (1963) reported that in *Vicia faba* leaves, both the magnitude of initial stomatal opening and the time required to reverse the effect increased with leaf water potential.

The positive relationship between pre-cut water status (assuming higher water potentials in leaves in higher humidity) and the time it took stomata to begin closing was also observed in the current study, but the relationship between magnitude of initial stomatal opening and VPD treatment was more complicated. Increases in *g*_s_ were significantly larger in the 1.25 than 2 kPa VPD treatment (*p* = 0.049). Because of the large variability of the *g*_s_ response in the 0.5 kPa VPD treatment, the average stomatal response in this treatment did not differ significantly from either the 2 or the 1.25 kPa VPD treatments. In fact, average *g*_s_ change in leaves of the 0.5 kPa VPD treatment was not statistically different from 0 (**Table 1**). This could be explained if stomata in the high humidity (low VPD) treatment were fully open at steady state. Guard cells may not have been able to open further, even with the pull of subsidiary epidermal cells as ψ_L_ decreased.

### RAPID RECOVERY OF *A* AND *E*

Although sunflower leaves have two large veins in addition to the mid-rib, we expected that severing of the mid-rib would disrupt water flow near the cut enough to increase the distance water must travel via non-vascular pathways of greater hydraulic resistance. This larger resistance would decrease *g*_s_ and *A*. However, leaves in all treatments exhibited rapid recovery, with *E* and *A* returning to within 10% of steady state (pre-cut) values within 15 min after severing the primary leaf vein. The recovery of *E* in the first 900 s after the vein was cut suggests that the hydraulic conductance of alternate flow paths in sunflower was sufficient to restore transpiration. Nardini et al. (2001) found high redundancy in leaves of *Prunus laurocerasus* such that the leaf mid-rib contributed relatively little to overall leaf conductance. This primary vein redundancy was also shown by Sack et al. (2008) in other species with palmate, but not pinnate, venation. In addition, it should be noted that our observation of little to no effect on *A* may be influenced by the fact that our study was conducted at about one quarter full sunlight (far below light saturation) in order to improve the signal to noise for variable chlorophyll fluorescence measurements and in well watered plants. Conditions that would maximize CO_2_ uptake or water loss might show lower apparent vascular redundancy.

The time at which transpiration rate reached its maximum could be interpreted as the time at which stomata reverse direction following their initial transient “wrong way” response to the vein cutting (i.e., the point at which re-hydration from alternative pathways begins). Re-hydration was found to occur sooner but with greater rate variability with higher VPD (**Figure 3**). This was probably because greater evaporative driving force increased the rate of water movement through alternative pathways. The increase in the variability of response recovery times with increasing VPD that was observed in the temperature response would be also expected if the mechanism at work were hydraulic. The spread between hydraulic flow rates across a leaf should increase with evaporative demand since differential conductance in different hydraulic pathways to leaf sub-areas would be magnified as the driving force increased.

### POSSIBLE SCENARIOS FOR PHOTOSYNTHETIC DECLINE

While the observed changes in *g*_s_ and *E* are consistent with stomatal responses to a perturbation of hydraulic architecture and water potential, the mechanism responsible for the transient decrease in *A* is more difficult to explain. Here we speculate briefly on what may have triggered the temporary photosynthetic decline when the observed change in *g*_s_ should have increased conductance of CO_2_ to photosynthetic tissues.

Photosynthesis can be metabolically limited at low ψ_L_ as a result of depressed ATP synthesis, ribulose-1,5-bisphosphate (RuBP) regeneration or Rubisco activity (Parry et al., 2002; Tang et al., 2002). However, impaired photosynthetic metabolism has been measured only when cell turgor loss is severe (Bota et al., 2004), which is unlikely to have been the case in this extremely transitory response. Increased non-photochemical quenching has been observed in sunflower under less severe water stress, but it has been associated with stomatal closure (Tezara et al., 2008), which did not occur in this study. Alternatively, the decline in *A* could have been triggered by the sudden decrease in xylem water potential transmitted to cells that must have caused the increase in *g*_s_ following the cut, if the mechanisms that caused each response occurred at different rates. However, data from the current study do not support this hypothesis. No relationship was found between the magnitudes of the photosynthetic and transpiration rate changes in leaf sub-areas; and in some sub-areas, only one parameter was found to respond to the cut. Leaf hydraulic architecture could cause differential hydraulic resistance between leaf xylem and non-vascular pathways (Tyree et al., 1981; Salleo et al., 2000; Trifilo et al., 2003) and could be responsible for the non-congruent responses of *A* and *g*_s_ observed. But heterobaric species (like sunflower) have bundle sheath extensions which can function as hydraulic conduits, directly connecting vascular tissue to the epidermis (McClendon, 1992; Pieruschka et al., 2005) and separating leaf regions between the extensions. This anatomy suggests that in sunflower the transpiration stream would be linked more directly to the epidermis than the photosynthetic mesophyll, a type of hydraulic partitioning that would buffer mesophyll cells from sudden ψ change (Zwieniecki et al., 2007) and would not have resulted in the immediate response of photosynthesis observed here if the trigger were water potential alone. Furthermore, in our experiment *g*_s_ was affected by VPD treatment but *A* was not, suggesting that *A* did not respond to the same hydraulic signal.

The observed spatial and temporal differences between the responses of *A* and *E* could occur if the cut produced two separate signals, both initiated by vein-cutting, which propagated independently across the leaf. Based on the similarity of the *A* response we observed and other *A* responses attributed to *E*-potential, the most likely second signal is an electrical signal, either initiated by the wound itself or hydraulically triggered. Electrical signals, propagated as VPs, have previously been detected in sunflower in response to flaming and light induction (Stankovic et al., 1998) and were found to directly follow sudden pressure increases in the xylem. In the present study, xylem ψ would have risen to 0 (atmospheric) at the site of the cut, regardless of transpiration rate and downstream resistance of cells and stomata. The magnitude and propagation of a resulting VP would have been similar for all VPD treatments. Once initiated, a VP can be transmitted to cells lateral to affected xylem through plasmodesmata and into the phloem pathway (Lautner et al., 2005), until the signal fades with time and distance from the point of stimulation. The short time frame, transience, and pattern (which radiated from main leaf veins) of the *A* response observed in sunflower, are consistent with the manner in which VPs travel through tissue (Fromm and Lautner, 2007).

Photosynthesis has also been found to decline following tissue injury where electrical potentials transmitted from the site of injury were thought to suppress photosynthesis by increasing the pH gradient and depressing enzyme activity in cell walls (Davies, 1987; Bulychev and Kamzolkina, 2006). A sudden and transient decline in photosynthesis was observed in leaflets of mimosa and poplar trees in response to flame induced wounding (Koziolek et al., 2003; Lautner et al., 2005). In both cases, the decline in photosynthesis was associated with a measured change in electrical potential, although it was inconclusive whether the signal was a direct result of wounding or initiated by a hydraulic signal (Malone, 1994). This issue was recently revisited by Gallé et al. (2013) in soybean and they found that the electrical signal was much more clearly linked to *A* than *g*_s_. They also showed that declines in *g*_m_ were closely correlated with declines in *A*, suggesting that CO_2_ limitation of *A* was occurring due to decreases in *g*_m_ rather than *g*_s_.

## Conflict of Interest Statement

The authors declare that the research was conducted in the absence of any commercial or financial relationships that could be construed as a potential conflict of interest.

## References

[B1] Bota J. Medrano H. Flexas J. (2004). Is photosynthesis limited by decreased Rubisco activity and RuBP content under progressive water stress? *New Phytol.* 162 671–681 10.1111/j.1469-8137.2004.01056.x 33873761

[B2] Buckley T. N. (2005). The control of stomata by water balance. *New Phytol.* 168 275–292 10.1111/j.1469-8137.2005.01543.x 16219068

[B3] Bulychev A. A. Kamzolkina N. A. (2006). Effect of action potential on photosynthesis and spatially distributed H^+^ fluxes in cells and chlorophasts of *Chara corallina*. *Russ. J. Plant Physiol.* 53 1–9 10.1134/S1021443706010018

[B4] Darwin F Pertz D. F. M. (1911). On a new method of estimating the aperture of stomata. *Proc. R. Soc. Lond. B Biol. Sci.* 84 136–154 10.1098/rspb.1911.0058

[B5] Davies E. (1987). Action potentials as multifunctional signals in plants: a unifying hypothesis to explain apparently disparate wound responses. *Plant Cell Environ.* 10 623–631 10.1111/j.1365-3040.1987.tb01844.x

[B6] Eckstein J. Artsaenko O. Conrad U. Peiker M. Beyschlag W. (1998). Abscisic acid is not necessarily required for the induction of patchy stomatal closure. *J. Exp. Bot.* 49 611–616

[B7] Farquhar G. D. Sharkey T. D. (1982). Stomatal conductance and photosynthesis. *Annu. Rev. Plant Physiol.* 33 317–345 10.1146/annurev.pp.33.060182.001533

[B8] Fromm J. Lautner S. (2007). Electrical signals and their physiological significance in plants. *Plant Cell Environ.* 30 249–257 10.1111/j.1365-3040.2006.01614.x 17263772

[B9] Gallé A. Lautner S. Flexas J. Ribas-Carbo M. Hanson D. Roesgen J. (2013). Photosynthetic responses of soybean (*Glycine max* L.) to heat-induced electrical signaling are predominantly governed by modifications of mesophyll conductance for CO_2_. *Plant Cell Environ*. 36 542–552 10.1111/j.1365-3040.2012.02594.x 22897236

[B10] Iwanoff L. (1928). Zur Methodik der Transpirationsbestimmung am Standort. *Ber. Dtsch. Bot. Ges.* 46 306–310

[B11] Jones H. G. Sutherland R. A. (1991). Stomatal control of xylem embolism. *Plant Cell Environ.* 14 607–612 10.1111/j.1365-3040.1991.tb01532.x

[B12] Koziolek C. Grams T. E. E. Schreiber U. Matyssek R. Fromm J. (2003). Transient knockout of photosynthesis mediated by electrical signals. *New Phytol.* 161 715–722 10.1111/j.1469-8137.2004.00985.x 33873726

[B13] Lange O. L. Losch R. Schulze E. D. Kappen L. (1971). Responses of stomata to changes in humidity. *Planta* 100 76–86 10.1007/BF00386887 24488104

[B14] Lautner S. Grams T. E. E. Matyssek R. Fromm J. (2005). Characteristics of electrical signals in poplar and responses in photosynthesis. *Plant Physiol.* 138 2200–2209 10.1104/pp.105.064196 16040648 PMC1183407

[B15] Lawson T. Weyers J. Brook R. A. (1998). The nature of heterogeneity in the stomatal behaviour of *Phaseolus vulgaris* L. primary leaves. *J. Exp. Bot.* 49 1387–1395

[B16] Malone M. (1994). Wound-induced hydraulic signals and stimulus transmission in *Mimosa pudica* L. *New Phytol.* 128 49–56 10.1111/j.1469-8137.1994.tb03985.x 33874540

[B17] McClendon J. H. (1992). Photographic survey of the occurrence of bundle sheath extensions in deciduous dicots. *Plant Physiol.* 99 1677–1679 10.1104/pp.99.4.1677 16669090 PMC1080680

[B18] Meinzer F. C. (2002). Co-ordination of vapour and liquid phase water transport properties in plants. *Plant Cell Environ.* 25 265–274 10.1046/j.1365-3040.2002.00781.x 11841669

[B19] Mencuccini M. Comstock J. (1999). Variability in hydraulic architecture and gas exchange of common bean (*Phaseolus vulgaris*) cultivars under well-watered conditions: interactions with leaf size. *Aust. J. Plant Physiol.* 26 115–124 10.1071/PP98137

[B20] Messinger S. M. Buckley T. N. Mott K. A. (2006). Evidence for involvement of photosynthetic processes in the stomatal response to CO_2_. *Plant Physiol.* 140 771–778 10.1104/pp.105.073676 16407445 PMC1361342

[B21] Mott K. A. Buckley T. N. (1998). Stomatal heterogeneity. *J. Exp. Bot.* 49 407–417

[B22] Mott K. A. Franks P. J. (2001). The role of epidermal turgor in stomatal interactions following a local perturbation in humidity. *Plant Cell Environ.* 24 657–662 10.1046/j.0016-8025.2001.00705.x

[B23] Mott K. A. Parkhurst D. F. (1991). Stomatal responses to humidity in air and helox. *Plant Cell Environ.* 14 509–515 10.1111/j.1365-3040.1991.tb01521.x

[B24] Nardini A. Salleo S. (2003). Effects of the experimental blockage of the major veins on hydraulics and gas exchange of *Prunus laurocerasus* L. leaves*. J. Exp. Bot.* 54 1213–1219 10.1093/jxb/erg130 12654872

[B25] Nardini A. Tyree M. T. Salleo S. (2001). Xylem Cavitation in the leaf of *Prunus laurocerasus* and its impact on leaf hydraulics. *Plant Physiol.* 125 1700–1709 10.1104/pp.125.4.1700 11299351 PMC88827

[B26] Parry M. A. J. Andralojc P. J. Khan S. Lea P. J. Keys A. J. (2002). Rubisco activity: effects of drought stress. *Ann. Bot.* 89 833–839 10.1093/aob/mcf103 12102509 PMC4233807

[B27] Pieruschka R. Schurr U. Jahnke S. (2005). Lateral gas diffusion inside leaves. *J. Exp. Bot.* 56 857–864 10.1093/jxb/eri072 15668225

[B28] Raschke K. (1970). Leaf hydraulic system: rapid epidermal and stomatal responses to changes in water supply. *Science* 167 189–191 10.1126/science.167.3915.189 17754137

[B29] Sack L. Cowan P. D. Jaikumar N. Holbrook N. M. (2003). The “hydrology” of leaves: co-ordination of structure and function in temperate woody species. *Plant Cell Environ.* 26 1343–1356 10.1046/j.0016-8025.2003.01058.x

[B30] Sack L. Dietrich E. M. Streeter C. M. Sanchez-Gomez D. Holbrook N. M. (2008). Leaf palmate venation and vascular redundancy confer tolerance of hydraulic disruption. *Proc. Natl. Acad. Sci. U.S.A.* 105 1567–1572 10.1073/pnas.0709333105 18227511 PMC2234185

[B31] Sack L. Holbrook N. M. (2006). Leaf hydraulics. *Annu. Rev. Plant Biol.* 57 361–381 10.1146/annurev.arplant.56.032604.144141 16669766

[B32] Saliendra N. Z. Sperry J. S. Comstock J. P. (1995). Influence of leaf water status on stomatal response to humidity, hydraulic conductance, and soil drought in *Betula occidentalis*. *Planta* 357–366 10.1007/BF00201396

[B33] Salleo S. Nardini A. Pitt F. Lo Gullo M. A. (2000). Xylem caviation and hydraulic control of stomatal conductance in Laurel (*Laurus nobilis* L.). *Plant Cell Environ.* 23 71–79 10.1046/j.1365-3040.2000.00516.x

[B34] Shimazaki K. I. Doi M. Assmann S. M. (2007). Light regulation of stomatal movement. *Annu. Rev. Plant Biol.* 58 219–247 10.1146/annurev.arplant.57.032905.105434 17209798

[B35] Sommerville K. E. Gimeno T. E. Ball M. C. (2010). Primary nerve (vein) density influences spatial heterogeneity of photosynthetic response to drought in two *Acacia* species. *Funct. Plant Biol.* 37 840–848 10.1071/FP10062

[B36] Sperry J. S. Alder N. N. Eastlack S. E. (1993). The effect of reduced hydraulic conductance on stomatal conductance and xylem cavitation. *J. Exp. Bot.* 44 1075–1082 10.1093/jxb/44.6.1075

[B37] Sperry J. S. Pockman W. T. (1993). Limitation of transpiration by hydraulic conductance and xylem cavitation in *Betula occidentalis*. *Plant Cell Environ.* 16 279–287 10.1111/j.1365-3040.1993.tb00870.x

[B38] Stankovic B. Witters D. L. Zawadzki T. Davies E. (1998). Action potentials and variation potentials in sunflower: an analysis of their relationships and distinguishing characteristics. *Physiol. Plant* 103 51–58 10.1034/j.1399-3054.1998.1030107.x

[B39] Tang A. C. Kawamitsu Y. Kanechi M. Boyer J. S. (2002). Photosynthetic oxygen evolution at low water potential in leaf discs lacking an epidermis. *Ann. Bot.* 89 861–870 10.1093/aob/mcf081 12102512 PMC4233803

[B40] Terashima I. (1992). Anatomy of non-uniform leaf photosynthesis. *Photosynth. Res.* 31 195–212 10.1007/BF00035537 24408060

[B41] Tezara W. Driscoll S. Lawlor D. W. (2008). Partitioning of photosynthetic electron flow between CO_2_ assimilation and O_2_ reduction in sunflower plants under water deficit. *Photosynthetica* 46 127–134 10.1007/s11099-008-0020-1

[B42] Trifilo P. Gasco A. Raimondo F. Nardini A. Salleo S. (2003). Kinetics of recovery of leaf hydraulic conductance and vein functionality from cavitation-induced embolism in sunflower. *J. Exp. Bot.* 54 2323–2330 10.1093/jxb/erg259 14504300

[B43] Tyree M. T. Sperry J. S. (1988). Do woody plants operate near the point of catastrophic xylem dysfunction caused by dynamic water stress?: answers from a model. *Plant Physiol.* 88 574–580 10.1104/pp.88.3.574 16666351 PMC1055627

[B44] Tyree M. T. Cruizat P. Benis M. Logullo M. A. Salleo S. (1981). The kinetics of rehydration of detached sunflower leaves from different initial water deficits. *Plant Cell Environ*. 4 309–317 10.1111/1365-3040.ep11604553

[B45] West J. D. Peak D. Peterson J. Q. Mott K. A. (2005). Dynamics of stomatal patches for a single surface of *Xanthium strumarium* L. leaves observed with fluorescence and thermal images. *Plant Cell Environ.* 28 633–641 10.1111/j.1365-3040.2005.01309.x

[B46] Willis A. J. Yemm E. W. Balasubramaniam S. (1963). Transpiration phenomena in detached leaves. *Nature* 199 265–266 10.1038/199265a0

[B47] Yang S. D. Tyree M. T. (1994). Hydraulic architecture of *Acer saccharum* and *A. rubrum:* comparison of branches to whole trees and the contribution of leaves to hydraulic resistance. *J. Exp. Bot.* 45 179–186 10.1093/jxb/45.2.179

[B48] Zwieniecki M. A. Brodribb T. J. Holbrook N. M. (2007). Hydraulic design of leaves: insights from rehydration kinetics. *Plant Cell Environ.* 30 910–921 10.1111/j.1365-3040.2007.001681.x 17617819

